# Evaluating performance of health care facilities at meeting HIV-indicator reporting requirements in Kenya: an application of K-means clustering algorithm

**DOI:** 10.1186/s12911-020-01367-9

**Published:** 2021-01-06

**Authors:** Milka Bochere Gesicho, Martin Chieng Were, Ankica Babic

**Affiliations:** 1 Department of Information Science and Media Studies, University of Bergen, Bergen, Norway; 2 Vanderbilt University Medical Center, Nashville, USA; 3 Department of Biomedical Engineering, Linköping University, Linköping, Sweden; 4 Institute of Biomedical Informatics, Moi University, Eldoret, Kenya

**Keywords:** K-means clustering, Completeness, Timeliness, Performance, DHIS2

## Abstract

**Background:**

The ability to report complete, accurate and timely data by HIV care providers and other entities is a key aspect in monitoring trends in HIV prevention, treatment and care, hence contributing to its eradication. In many low-middle-income-countries (LMICs), aggregate HIV data reporting is done through the District Health Information Software 2 (DHIS2). Nevertheless, despite a long-standing requirement to report HIV-indicator data to DHIS2 in LMICs, few rigorous evaluations exist to evaluate adequacy of health facility reporting at meeting completeness and timeliness requirements over time. The aim of this study is to conduct a comprehensive assessment of the reporting status for HIV-indicators, from the time of DHIS2 implementation, using Kenya as a case study.

**Methods:**

A retrospective observational study was conducted to assess reporting performance of health facilities providing any of the HIV services in all 47 counties in Kenya between 2011 and 2018. Using data extracted from DHIS2, K-means clustering algorithm was used to identify homogeneous groups of health facilities based on their performance in meeting timeliness and completeness facility reporting requirements for each of the six programmatic areas. Average silhouette coefficient was used in measuring the quality of the selected clusters.

**Results:**

Based on percentage average facility reporting completeness and timeliness, four homogeneous groups of facilities were identified namely: best performers, average performers, poor performers and outlier performers. Apart from blood safety reports, a distinct pattern was observed in five of the remaining reports, with the proportion of best performing facilities increasing and the proportion of poor performing facilities decreasing over time. However, between 2016 and 2018, the proportion of best performers declined in some of the programmatic areas. Over the study period, no distinct pattern or trend in proportion changes was observed among facilities in the average and outlier groups.

**Conclusions:**

The identified clusters revealed general improvements in reporting performance in the various reporting areas over time, but with noticeable decrease in some areas between 2016 and 2018. This signifies the need for continuous performance monitoring with possible integration of machine learning and visualization approaches into national HIV reporting systems.

## Background

The Human Immunodeficiency Virus (HIV) epidemic remains a challenge globally with highest infected numbers found in countries in East and Southern Africa, which accounted for an estimated 20.7 million infected individuals in 2019 [1]. Efforts to eradicate the HIV epidemic have seen affected countries in low-middle-income-countries (LMICs) receive substantial support from donors and multilateral global organizations in order to scale-up HIV services such as antiretroviral therapy (ART), prevention of mother-to-child transmission (PMTCT) of HIV, and HIV testing and counselling (HTC) [2]. This has brought about the need to strengthen strategic information on HIV. Health Management Information Systems (HMIS), through better data quality, improves decision-making such as informing policy, measuring program effectiveness, advocacy and resource allocation [3]. Ministries of Health (MoH) and donor organizations require facilities providing HIV services to report several aggregated HIV-indicators as part of Monitoring and Evaluation (M&E) program [4, 5].

The scale-up of HIV services has contributed to strengthening of HMIS in many low-middle-income-countries, resulting in improved availability of routinely generated HIV aggregate indicator data from health facilities to the national level [6]. HIV indicator data typically comes from aggregation of monthly reports generated by various facilities that are collated in summary forms and submitted to an aggregate-level HMIS or reporting system [6]. One such national-level data aggregation system is the District Health Information Software Version 2 (DHIS2), which has been adopted by many LMICs [7].

Aggregate data stored in systems such as DHIS2 are only as good as their quality [8]. Therefore, the ability to report complete, accurate and timely data by HIV care providers and other entities is a key aspect in monitoring trends in HIV care. Various approaches to evaluating data quality have been proposed such as desk reviews, data verification or system assessments across the following data quality dimensions; completeness, timeliness, internal consistency of reported data, external comparisons and external consistency of population data [9]. Evaluations on quality of indicator reporting leveraging some of these approaches have previously been conducted within DHIS2 based on various data quality dimensions [10–14].Nonetheless, despite a long-standing requirement to report HIV indicator data to DHIS2 in LMICs, few rigorous evaluations exist to evaluate adequacy of health facility reporting at meeting completeness and timeliness requirements over time.

Rigorous reporting by facilities into DHIS2 over time is imperative to identify changes in trends and implement timely interventions [14]. In this study, we aim to leverage on machine learning algorithms as well as data visualization approaches to conduct a comprehensive assessment of the reporting performance for HIV-indicators at the national-level by facilities using completeness and timeliness indicators, with Kenya as a case study.

## Methods

### Related works

Table 1 illustrates some of the related studies that have extracted data from DHIS2 in order to evaluate performance at meeting the various dimensions of data quality. In addition, data from these studies was gathered from various time periods as well as various areas within health care such as malaria.

**Table 1:** Summary of some of the related works evaluating various dimensions of data quality

Studies	Dimensions evaluated					
	Facility reporting completeness	Indicator data completeness	Timeliness	Internal consistency	External consistency	Summary
Bhattacharya et al. [10]	X	X	X	X	X	Extracted priority maternal and neonatal health indicators Data gathered from July 2016 to June 2017
Githinji et al. [11]	X	X	–	–	–	Extracted malaria indicator data Data gathered from 2011–2015
Adokiya et al. [12]	X	–	X	–	–	Extracted disease surveillance and response reports Data gathered from 2012 and 2013
Nisingizwe et al. [14]	X	X	–	X	–	Extracted health management information systems data for selected indicators Data gathered from 2008–2012
Kiberu et al. [13]	X	–	X	–		Extracted inpatient and outpatient data Data gathered from 2011/12 and after 2012/13

Whereas our study focused on facility reporting completeness and timeliness of HIV-indicators for the period of 2011 to 2018, the difference compared with the other studies is leveraging of the k-means clustering algorithm.

### Study setting

This study was conducted in Kenya, a sub-Saharan country made up of 47 counties. Administratively, the health care service delivery system has six levels, namely: community, dispensary, health center, district hospital, provincial hospital, and national referral hospital [15]. Kenya adopted the DHIS2 in 2011 at the national level for aggregation of health data across different levels of the health system [16, 17].

### Study design

A retrospective observational study was conducted in order to identify reporting performance over time by health facilities in meeting completeness and timeliness reporting requirements.

### Data source

Data for facilities reporting completeness and timeliness between the years 2011 and 2018 were extracted from the DHIS2 in Kenya. DHIS2 is a web-based open-source health management information system developed for purposes of collecting aggregate level data routinely generated across health facilities in various countries [7, 16]. DHIS2 also supports various activities and contains modules for processes such as data management and analytics, which contain features for data visualization, charts, pivot tables and dashboards [18]. It is also currently in use by ministries of health in over 70 countries [19]. In Kenya, DHIS2 was rolled out nationally in the year 2011 [16]. Reporting completeness and timeless data were extracted from Kenya’s DHIS2 for all facilities in all the 47 counties in Kenya. Systematic procedures were used in cleaning the data using a generic five-step approach as outlined in Gesicho et al. [20]. Data used were only for facilities that offered one or more of the outlined HIV services that required reporting, namely: (1) HIV testing and counselling (HTC), (2) Prevention of Mother to Child Transmission (PMTCT), (3) Care and Treatment (CRT), (4) Voluntary Medical Male Circumcision (VMMC), (5) Post-Exposure Prophylaxis (PEP) and (6) Blood Safety (BS). These data were derived based on the MOH 731 Comprehensive HIV/AIDS facility-reporting form, which is the major monthly HIV summary report required by the MOH in Kenya and used by health facilities for reporting of HIV-indicators into DHIS2. It is worth noting that health facilities are not required to report on indicators for all the six programmatic areas, but only those for which they provide services. As such, there are variations in number of facilities (n) in the various programmatic reporting areas.

## Measures

### Facility reporting completeness and timeliness

Percentage completeness in facility reporting is calculated automatically within Kenya’s DHIS2 and is defined as the number of actual monthly reports received divided by the expected number of reports in a given year. Percentage timeliness in facility reporting is also calculated automatically within Kenya’s DHIS2 and is defined as the number of actual monthly reports received on time (by the 15th of every month) divided by the expected number of reports in a given year. Facility reporting completeness and timeliness were selected as indicators for assessing reporting performance as they were readily available within DHIS2 for the eight year period covered by the study.

### Outcome measures

The primary outcome of interest consisted of identifying the performance in reporting by health facilities over time (2011–2018), with facilities put into various performance clusters and performance evaluated in the various programmatic areas.

### Data analysis

K-means algorithm was preferred due to its efficiency and suitability in pattern recognition, its simplicity, ease of implementation as well as its empirical success [21]. K-means algorithm is a non-hierarchical procedure where *k* represents the number of clusters, which need to be specified prior to any clustering [22]. Given that K-means algorithm uses unsupervised learning, the idea was to group the health facilities into *k* homogeneous groups based on their performance in completeness and timeliness, in each of the six programmatic areas for each of the study years. Based on the data set and purpose of this study, we used the average silhouette coefficient, which is an intrinsic method of measuring the quality of a cluster [23]. The average value of the silhouette coefficient ranges between − 1 (least preferable value indicating poor structure) and + 1 (most preferable value indicating good structure). According to Kaufman and Rousseeuw, average silhouette measure that is greater than + 0.5 indicates reasonable partitioning of data, whereas greater than + 0.7 indicates a strong partitioning [24]. On the other hand average silhouette measures lower than + 0.5 indicate a weak or artificial partitioning, whereas below + 0.2 indicates no clusters can be exhibited from the data [24].

In order to determine the number of clusters (*k*) to be generated, the Euclidean distance measure was applied and *k* was specified within a set of values [21, 25]. The range of *k* values was then iteratively re-run with two values of k (k = 3 and k = 4) and inspecting the average corresponding silhouette values [26].

**Fig. 1:**
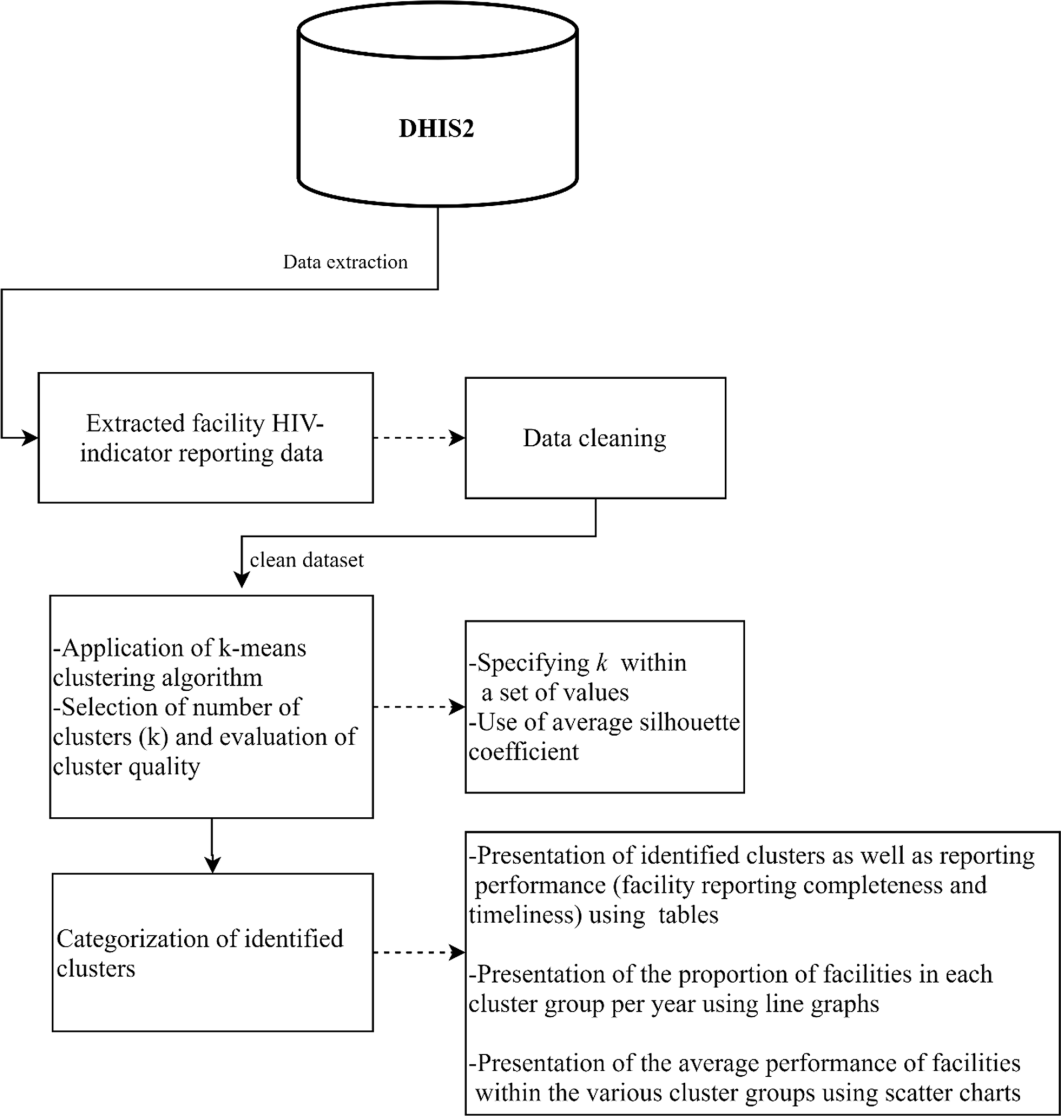
Summary of methods

The proportion of facilities in the various cluster groups was then determined by calculating the percentage number of facilities in a particular cluster group out of the total facilities in that particular year. To illustrate the average performance of facilities within the various cluster groups, we developed a scatter chart visualization using Tableau [27]. In addition, HTC programmatic area was used as an illustrative example for the visualization, given that it is one of the most reported programmatic areas. Figures and tables were developed using Microsoft Word and Excel (Microsoft Office Version 18.2008.12711.0). All analyses were performed using SPSS [28]. A summary of the methods is illustrated in Fig. 1.

## Results

Results from the silhouette coefficient average measures for each reporting area are presented in Table 2. The results ascertain that the average silhouette values for both k = 3 and k = 4 produce reasonable to strong partitioning except for 2011 under CRT where the values for k = 3 where below 0.5, hence k = 4 was used in this case. Therefore, based on method criteria and interpretability of the data set, either k = 3 and k = 4 were used where reasonable to strong partitions were identified in the average silhouette measures. As such, k = 4 was used when more variation could be provided in the data from four clusters, and k = 3 was used when three clusters provided more variation than four clusters. For VMMC and PEP programmatic areas, the number of health facilities was not enough to conduct cluster analysis in the year 2011.

**Table 2:** Average of the Silhouette of a k-means clustering when k = 3 and k = 4

Average silhouette measures								
HTC			PMTCT			CRT		
Year	K = 3	K = 4	Year	K = 3	K = 4	Year	K = 3	K = 4
2011	0.800	0.775	2011	0.674	0.706	2011	**0.368**	0.582
2012	0.526	0.563	2012	0.585	0.588	2012	0.556	0.599
2013	0.659	0.648	2013	0.654	0.632	2013	0.637	0.618
2014	0.669	0.669	2014	0.676	0.666	2014	0.692	0.663
2015	0.737	0.709	2015	0.649	0.711	2015	0.710	0.705
2016	0.749	0.754	2016	0.791	0.774	2016	0.708	0.710
2017	0.685	0.673	2017	0.699	0.677	2017	0.696	0.700
2018	0.593	0.714	2018	0.689	0.707	2018	0.654	0.701

VMMC			PEP			BS		
Year	K = 3	K = 4	Year	K = 3	K = 4	Year	K = 3	K = 4
2011	^a^	^a^	2011	0.704	0.679	2011	^a^	^a^
2012	1.00	^b^	2012	0.593	0.605	2012	0.734	0.730
2013	0.64	0.669	2013	0.639	0.629	2013	0.732	0.687
2014	0.634	0.661	2014	0.675	0.667	2014	0.712	0.650
2015	0.733	0.681	2015	0.682	0.673	2015	0.617	0.641
2016	0.708	0.699	2016	0.696	0.665	2016	0.719	0.680
2017	0.765	0.733	2017	0.621	0.611	2017	0.577	0.637
2018	0.657	0.636	2018	0.650	0.673	2018	0.610	0.607

^a^There are not enough valid cases to conduct the specified cluster analysis

^b^In the data, there is insufficient variation to honor the four clusters specified. The number of clusters is reduced to 3

The four clusters were characterized based on health facility performance as follows:

- *Best performers* This cluster consisted of health facilities that had the highest percentage in reporting completeness and timeliness in a particular reporting year.
- *Average performers* This cluster consisted of health facilities that had lower percentage in reporting completeness and timeliness compared to best performers in a particular year.
- *Poor performers* This cluster consisted of health facilities with lowest percentage in reporting completeness and timeliness in a particular year.
- *Outlier performers* This cluster consisted of health facilities with high percentage in completeness compared to average performers, but with low percentage in timeliness in that particular year.

Performance was therefore categorized per year by cluster. As such, the average percentage reporting completeness and timeliness for a particular cluster group may vary by year. It is worth noting that there were no clusters with low completeness and high timeliness as reports cannot be on time if they were not submitted in the first place. Detailed results by cluster for each reporting programmatic area are outlined below.

**Fig. 2:**
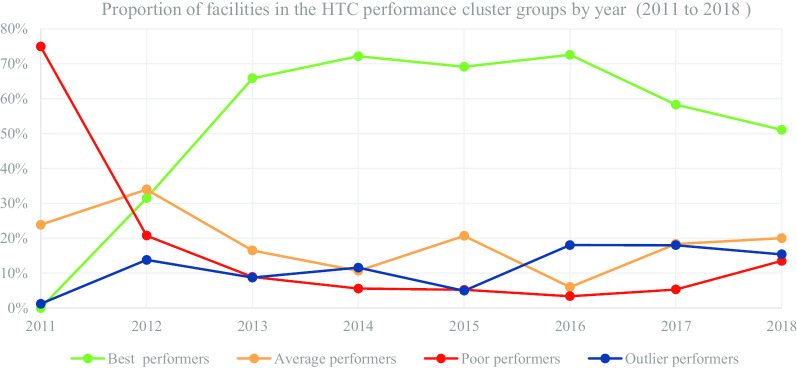
HTC performance trend based on proportion of facilities by year

In Table 3 and Fig. 2, we present the segmentation of facilities based on performance cluster groups according to the HTC programmatic area. As such, Table 3 includes the average percentage for facility reporting completeness and timeliness for each cluster group in HTC for the number of facilities (n) in a particular year.

**Table 3:** HIV testing and counselling (HTC)-health facility (n) segmentation based on performance clusters

Year	2011				2012			
Cluster group	Best n = 0	Average n = 177	Poor n = 556	Outlier n = 9	Best n = 1206	Average n = 1301	Poor n = 794	Outlier n = 528
MOH 731-1 HTC completeness	0.00	24.49	13.07	91.67	90.08	55.30	25.68	86.75
MOH 731-1 HTC timeliness	0.00	16.63	2.91	21.30	80.47	45.65	16.11	46.17

Year	2013				2014			
Cluster group	Best n = 3219	Average n = 806	Poor n = 437	Outlier n = 427	Best n = 3837	Average n = 568	Poor n = 297	Outlier n = 615
MOH 731-1 HTC completeness	96.73	68.77	32.96	89.86	98.18	73.07	33.75	95.94
MOH 731-1 HTC timeliness	89.55	57.33	21.63	43.00	92.96	62.42	23.02	54.06

Year	2015				2016			
Cluster group	Best n = 3916	Average n = 1172	Poor n = 296	Outlier n = 282	Best n = 4376	Average n = 362	Poor n = 205	Outlier n = 1089
MOH 731-1 HTC completeness	99.40	88.30	34.57	93.09	99.34	69.15	31.47	91.29
MOH 731-1 HTC timeliness	96.33	71.71	27.45	33.45	95.89	51.07	20.29	74.04

Year	2017				2018			
Cluster group	Best n = 3698	Average n = 1164	Poor n = 338	Outlier n = 1143	Best n = 3403	Average n = 1334	Poor n = 899	Outlier n = 1026
MOH 731-1 HTC completeness	97.98	64.47	32.69	94.20	88.48	52.68	26.87	77.35
MOH 731-1 HTC timeliness	93.92	57.04	23.59	64.33	86.93	48.84	22.98	64.65

Figure 2 consists of a graphical presentation of the proportion of facilities in each cluster group per year for HTC. Based on performance trends presented in Fig. 2, the proportion of best performing facilities accounted for 72.55% in 2016, which was a progressive increase from 31.50% in 2012. Nonetheless, in 2017 and 2018 the proportion of best performing facilities accounted for 58.30% and 51.08% respectively, which was a progressive decrease from 72.55% in 2016. On the other hand, the proportion of poor performing facilities accounted for 3.40% in 2016, which was a progressive decrease from 74.93% in 2011. However, the proportion of poor performing facilities accounted for 13.49% in 2018, which was a progressive increase from 3.40% in 2016.

The proportion of average and outlier performing facilities varied in the different years with no steady trend. Nonetheless, in the latter years, the proportion of average performing facilities accounted for 20.02% in 2018, which was a progressive increase from 6.00% in 2016. On the other hand, proportion of outlier performers accounted for 15.40% in 2018, which was a decrease from 18.02% in 2017.

**Fig. 3:**
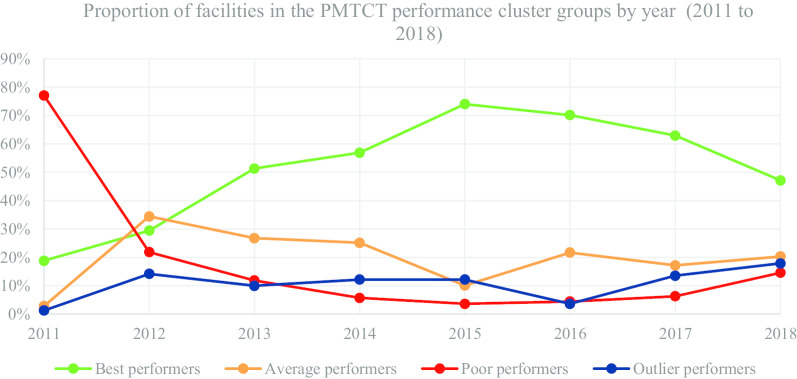
PMTCT performance trend based on proportion of facilities by year

In Table 4 and Fig. 3, we present the segmentation of facilities based on performance cluster groups according to the PMTCT programmatic area. As such, Table 4 includes the average percentage for facility reporting completeness and timeliness for each cluster group in PMTCT for the number of facilities (n) in a particular year.

**Table 4:** Prevention of Mother to Child Transmission (PMTCT)—health facility (n) segmentation based on performance clusters

Year	2011				2012			
Cluster group	Best n = 132	Average n = 20	Poor n = 541	Outlier n = 9	Best n = 1052	Average n = 1230	Poor n = 782	Outlier n = 508
MOH 731-2 PMTCT completeness	21.67	38.32	12.91	91.67	90.03	55.51	26.09	85.65
MOH 731-2 PMTCT timeliness	18.64	4.58	2.81	18.52	80.87	45.55	16.20	47.33

Year	2013				2014			
Cluster group	Best n = 2277	Average n = 1188	Poor n = 527	Outlier n = 444	Best n = 2737	Average n = 1210	Poor n = 277	Outlier n = 586
MOH 731-2 PMTCT completeness	97.73	84.02	37.19	85.98	98.61	89.43	37.03	96.26
MOH 731-2 PMTCT timeliness	92.11	63.53	26.11	29.70	92.31	59.29	24.02	14.54

Year	2015				2016			
Cluster group	Best n = 3785	Average n = 517	Poor n = 187	Outlier n = 625	Best n = 2732	Average n = 1156	Poor n = 237	Outlier n = 194
MOH 731-2 PMTCT completeness	98.84	75.61	30.34	98.13	99.43	90.03	37.95	89.32
MOH 731-2 PMTCT timeliness	91.22	61.72	21.97	38.76	95.42	72.36	25.98	38.46

Year	2017				2018			
Cluster group	Best n = 3456	Average n = 944	Poor n = 348	Outlier n = 744	Best n = 2685	Average n = 1259	Poor n = 832	Outlier n = 1018
MOH 731-2 PMTCT completeness	97.58	64.96	38.51	93.73	88.48	53.03	27.69	79.02
MOH 731-2 PMTCT timeliness	91.54	58.59	26.55	54.52	86.72	48.22	22.65	63.20

Figure 3 consists of a graphical presentation of the proportion of facilities in each cluster group per year for PMTCT. Based on performance trends presented in Fig. 3, the proportion of best performing facilities accounted for 74.01% in 2015, which was a progressive increase from 18.80% in 2011. Nonetheless, in 2018 the proportion of best performing facilities accounted for 47.15%, which was a progressive decrease from 74.01% in 2015. On the other hand, the proportion of poor performing facilities accounted for 3.66% in 2015, which was a progressive decrease from 77.07% in 2011. However, in 2018 the proportion of poor performing facilities accounted for 14.61%, which was a progressive increase from 3.66% in 2015.

The proportion of average and outlier performing facilities varied in the different years with no steady trend. Nonetheless, for the latter years, proportion of average performing facilities accounted for 20.34% in 2018, which was an increase from 17.19% in 2017. On the other hand, proportion of outlier performers accounted for 17.90% in 2018, which was an increase from 3.65% in 2016.

**Fig. 4:**
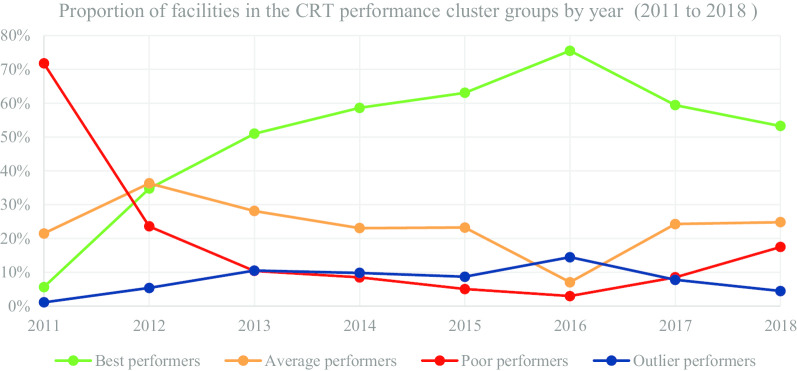
CRT performance trend based on proportion of facilities by year

In Table 5 and Fig. 4, we present the segmentation of facilities based on performance cluster groups according to the CRT programmatic area. As such, Table 5 includes the average percentage for facility reporting completeness and timeliness for each cluster group in CRT for the number of facilities (n) in a particular year.

**Table 5:** Care and Treatment (CRT)—health facility (n) segmentation based on performance clusters

Year	2011				2012			
Cluster group	Best n = 20	Average n = 76	Poor n = 254	Outlier n = 4	Best n = 634	Average n = 662	Poor n = 430	Outlier n = 98
MOH 731-3 care and treatment completeness	42.50	21.61	12.49	93.75	90.00	57.90	24.69	84.61
MOH 731-3 care and treatment timeliness	2.09	17.79	2.70	22.93	76.54	46.74	15.29	22.81

Year	2013				2014			
Cluster group	Best n = 1063	Average n = 587	Poor n = 217	Outlier n = 219	Best n = 1407	Average n = 554	Poor n = 204	Outlier n = 236
MOH 731-3 care and treatment completeness	97.67	81.29	31.24	90.05	98.67	87.86	34.51	94.53
MOH 731-3 care and treatment timeliness	90.81	59.82	19.79	24.03	92.01	62.55	27.03	24.65

Year	2015				2016			
Cluster group	Best n = 1647	Average n = 607	Poor n = 132	Outlier n = 227	Best n = 2171	Average n = 203	Poor n = 86	Outlier n = 416
MOH 731-3 care and treatment completeness	99.00	93.63	35.73	93.96	99.09	76.65	27.22	97.21
MOH 731-3 care and treatment timeliness	94.71	66.06	23.13	25.27	91.13	59.43	16.15	38.87

Year	2017				2018			
Cluster group	Best n = 1837	Average n = 750	Poor n = 264	Outlier n = 241	Best n = 1676	Average n = 781	Poor n = 550	Outlier n = 141
MOH 731-3 care and treatment completeness	98.82	92.41	43.10	95.74	86.94	55.13	26.68	71.65
MOH 731-3 care and treatment timeliness	94.22	65.41	32.70	27.61	81.75	50.71	23.26	21.37

Figure 4 consists of a graphical presentation of the proportion of facilities in each cluster group per year for CRT. Based on performance trends presented in Fig. 4, the proportion of best performing facilities accounted for 75.49% in 2016, which was a progressive increase from 5.65% in 2011. Nonetheless, in 2018 the proportion of best performing facilities accounted for 53.24%, which was a progressive decrease from 75.49% in 2016. On the other hand, the proportion of poor performing facilities accounted for 2.99% in 2016, which was a progressive decrease from 71.75% in 2011. However, in 2018 the proportion of poor performing facilities accounted for 17.47%, which was a progressive increase from 2.99% in 2016.

The proportion of average and outlier performing facilities varied in the different years with no steady trend. Nonetheless, for the latter years the proportion of average performing facilities accounted for 24.81% in 2018, which was an increase from 7.06% in 2016. On the other hand, proportion of outlier performers accounted for 4.48% in 2018, which was a progressive decrease from 14.46% in 2016.

**Fig. 5:**
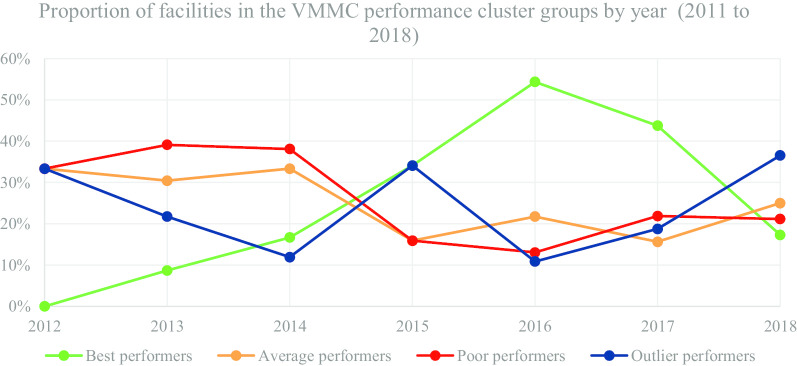
VMMC performance trend based on proportion of facilities by year

In Table 6 and Fig. 5, we present the segmentation of facilities based on performance cluster groups according to the VMMC programmatic area. As such, Table 6 includes the average percentage for facility reporting completeness and timeliness for each cluster group in VMMC for the number of facilities (n) in a particular year.

**Table 6:** Voluntary Medical Male Circumcision (VMMC)-health facility (n) segmentation based on performance clusters

Year	2012				2013			
Cluster group	Best n = 0	Average n = 2	Poor n = 2	Outlier n = 2	Best n = 2	Average n = 7	Poor n = 4	Outlier n = 5
MOH 731-4 VMMC completeness	0.00	17.00	8.00	8.00	54.50	35.57	13.89	51.80
MOH 731-4 VMMC timeliness	0.00	17.00	8.00	0.00	50.00	19.00	7.33	23.40

Year	2014				2015			
Cluster group	Best n = 7	Average n = 14	Poor n = 16	Outlier n = 5	Best n = 15	Average n = 7	Poor n = 7	Outlier n = 15
MOH 731-4 VMMC completeness	85.86	51.14	20.38	81.80	95.07	50.00	15.57	86.67
MOH 731-4 VMMC timeliness	81.14	39.36	13.00	36.60	88.38	42.86	14.43	62.20

Year	2016				2017			
Cluster group	Best n = 25	Average n = 10	Poor n = 7	Outlier n = 4	Best n = 28	Average n = 10	Poor n = 14	Outlier n = 12
MOH 731-4 VMMC completeness	97.12	67.60	17.86	70.75	92.61	52.40	17.79	86.83
MOH 731-4 VMMC timeliness	90.00	62.60	13.14	16.75	86.88	37.40	10.57	58.31

Year	2018							
Cluster group	Best n = 9	Average n = 13	Poor n = 11	Outlier n = 19				
MOH 731-4 VMMC completeness	85.73	43.94	19.09	61.58				
MOH 731-4 VMMC timeliness	81.11	36.15	16.36	55.26

Figure 5 consists of a graphical presentation of the proportion of facilities in each cluster group per year for VMMC. Based on performance trends presented in Fig. 5, the proportion of best performing facilities accounted for 54.35% in 2016, which was a progressive increase from 8.70% in 2013. Nonetheless, in 2018 the proportion of best performing facilities accounted for 17.31%, which was a progressive decrease from 54.35% in 2016. On the other hand, the proportion of poor performing facilities accounted for 13.04% in 2016, which was a progressive decrease from 39.13%% in 2013. However, in 2017 and 2018 the proportion of poor performing facilities accounted for 21.88% and 21.15%, which was a progressive increase from 13.04% in 2016.

The proportion of average and outlier performing facilities varied in the different years with no steady trend. Nonetheless, for the latter years, the proportion of average performing facilities accounted for 25.00% in 2018, which was an increase from 15.63% in 2017. On the other hand, proportion of outlier performers accounted for 36.54% in 2018, which was a progressive increase from 10.87% in 2016.

**Fig. 6:**
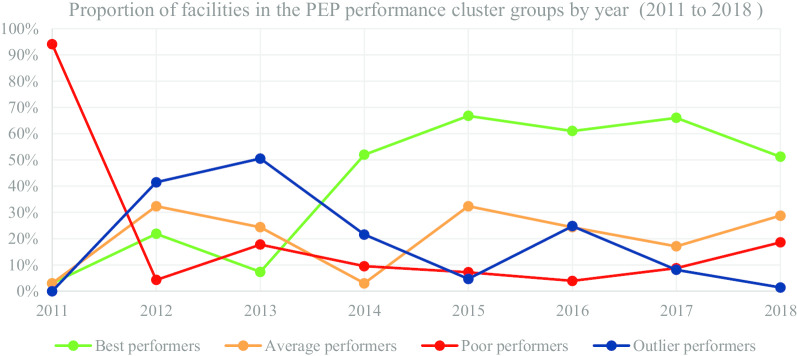
PEP performance trend based on proportion of facilities by year

In Table 7 and Fig. 6, we present the segmentation of facilities based on performance cluster groups according to the PEP programmatic area. As such, Table 7 includes the average percentage for facility reporting completeness and timeliness for each cluster group in PEP for the number of facilities (n) in a particular year.

**Table 7:** Post-Exposure Prophylaxis (PEP)-health facility (n) segmentation based on performance clusters

Year	2011				2012			
Cluster groups	Best n = 0	Average n = 2	Poor n = 63	Outlier n = 2	Best n = 173	Average n = 256	Poor n = 328	Outlier n = 34
MOH 731-5 post-exposure prophylaxis completeness	0.00	54.20	13.48	95.85	84.98	54.24	23.56	89.71
MOH 731-5 post-exposure prophylaxis timeliness	0.00	4.15	6.18	8.35	73.74	44.72	15.65	34.07

Year	2013				2014			
Cluster groups	Best n = 583	Average n = 281	Poor n = 205	Outlier n = 85	Best n = 677	Average n = 221	Poor n = 124	Outlier n = 281
MOH 731-5 post-exposure prophylaxis completeness	94.44	61.18	29.01	87.45	97.04	56.66	23.39	83.53
MOH 731-5 post-exposure prophylaxis timeliness	88.01	51.75	20.00	41.74	93.14	40.20	17.24	63.91

Year	2015				2016			
Cluster groups	Best n = 954	Average n = 305	Poor n = 103	Outlier n = 67	Best n = 953	Average n = 161	Poor n = 61	Outlier n = 387
MOH 731-5 post-exposure prophylaxis completeness	97.14	76.33	27.25	78.37	98.15	59.58	27.85	83.22
MOH 731-5 post-exposure prophylaxis timeliness	93.05	62.86	22.34	29.24	95.37	46.37	22.83	70.99

Year	2017				2018			
Cluster groups	Best n = 1031	Average n = 267	Poor n = 137	Outlier n = 127	Best n = 725	Average n = 407	Poor n = 263	Outlier n = 20
MOH 731-5 post-exposure prophylaxis completeness	95.73	66.51	38.29	90.02	85.04	54.06	24.07	80.50
MOH 731-5 post-exposure prophylaxis timeliness	91.35	59.21	28.23	54.50	82.38	49.99	20.32	36.46

Figure 6 consists of a graphical presentation of the proportion of facilities in each cluster group per year for PEP. Based on performance trends presented in Fig. 6, the proportion of best performing facilities accounted for 66.76% in 2015, which was a progressive increase from 2.99% in 2011. Nonetheless, in 2018 the proportion of best performing facilities accounted for 51.24%, which was a decrease from 66.01% in 2017. On the other hand, the proportion of poor performing facilities accounted for 3.91% in 2016, which was a progressive decrease from 17.76% in 2013. However, in 2018 the proportion of poor performing facilities accounted for 18.59%, which was a progressive increase from 3.91% in 2016.

The proportion of average and outlier performing facilities varied in the different years with no steady trend. Nonetheless, for the latter years the proportion of average performing facilities accounted for 28.76% in 2018, which was an increase from 17.09% in 2017. On the other hand, proportion of outlier performers accounted for 1.41% in 2018, which was a progressive decrease from 24.78% in 2016.

**Fig. 7:**
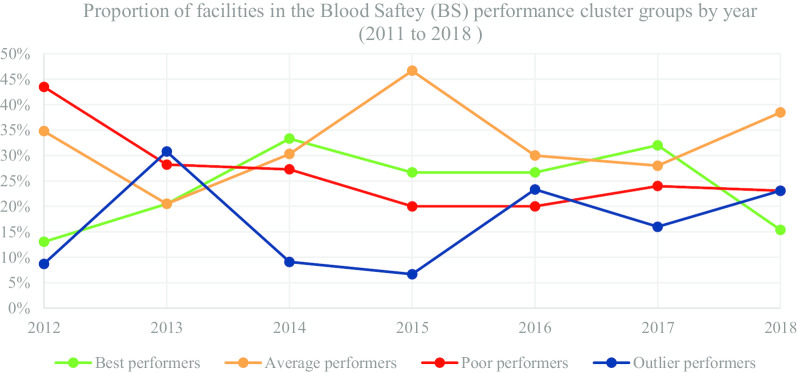
BS performance trend based on proportion of facilities by year

In Table 8 and Fig. 7, we present the segmentation of facilities based on performance cluster groups according to the BS programmatic area. As such, Table 8 includes the average percentage for facility reporting completeness and timeliness for each cluster group in BS for the number of facilities (n) in a particular year.

**Table 8:** Blood safety (BS)—health facility segmentation based on performance clusters

Year	2012				2013			
Cluster groups	Best n = 3	Average n = 8	Poor n = 10	Outlier n = 2	Best n = 8	Average n = 8	Poor n = 11	Outlier n = 12
MOH 731-6 blood safety completeness	69.67	43.75	18.30	100.00	94.88	37.50	15.82	75.75
MOH 731-6 blood safety timeliness	67.00	35.25	14.23	54.00	91.50	30.13	8.18	57.00

Year	2014				2015			
Cluster groups	Best n = 11	Average n = 10	Poor n = 9	Outlier n = 3	Best n = 8	Average n = 14	Poor n = 6	Outlier n = 2
MOH 731-6 blood safety completeness	95.55	67.60	47.33	97.33	87.38	62.43	22.17	58.00
MOH 731-6 blood safety timeliness	87.95	62.50	40.33	22.33	81.25	45.86	15.17	8.50

Year	2016				2017			
Cluster groups	Best n = 8	Average n = 9	Poor n = 6	Outlier n = 7	Best n = 8	Average n = 7	Poor n = 6	Outlier n = 4
MOH 731-6 blood safety completeness	94.88	69.56	47.33	27.14	83.25	56.00	26.33	79.25
MOH 731-6 blood safety timeliness	92.79	62.00	40.33	17.86	78.13	54.86	22.33	41.50

Year	2018							
Cluster groups	Best n = 2	Average n = 5	Poor n = 3	Outlier n = 3				
MOH 731-6 blood safety completeness	85.00	54.00	26.67	66.67				
MOH 731-6 blood safety timeliness	75.00	34.00	26.67	53.33

Figure 7 consists of a graphical presentation of the proportion of facilities in each cluster group per year for BS. Based on performance trends presented in Fig. 7, the proportion of best performing facilities accounted for 26.67% in 2015 and 2016, which was a decrease from 33.33% in 2014. Nonetheless, in 2018 the proportion of best performing facilities accounted for 15.38%, which was a decrease from 32.00% in 2017. On the other hand, the proportion of poor performing facilities accounted for 20.00% in 2015 and 2016, which was a progressive decrease from 43.48% in 2011. However, in 2017 the proportion of poor performing facilities accounted for 24.00%, which was an increase from 2016. For the latter years, the proportion of average performing facilities accounted for 28.00% in 2017 and 38.46% in 2018. On the other hand, proportion of outlier performers accounted for 16.00% in 2017 and 23.08% 2018. Nonetheless, there have been a general progressive decrease in facilities submitting BS indicators from 2013 to 2018.

### Scatter chart visualization of HTC performance clusters

**Fig. 8:**
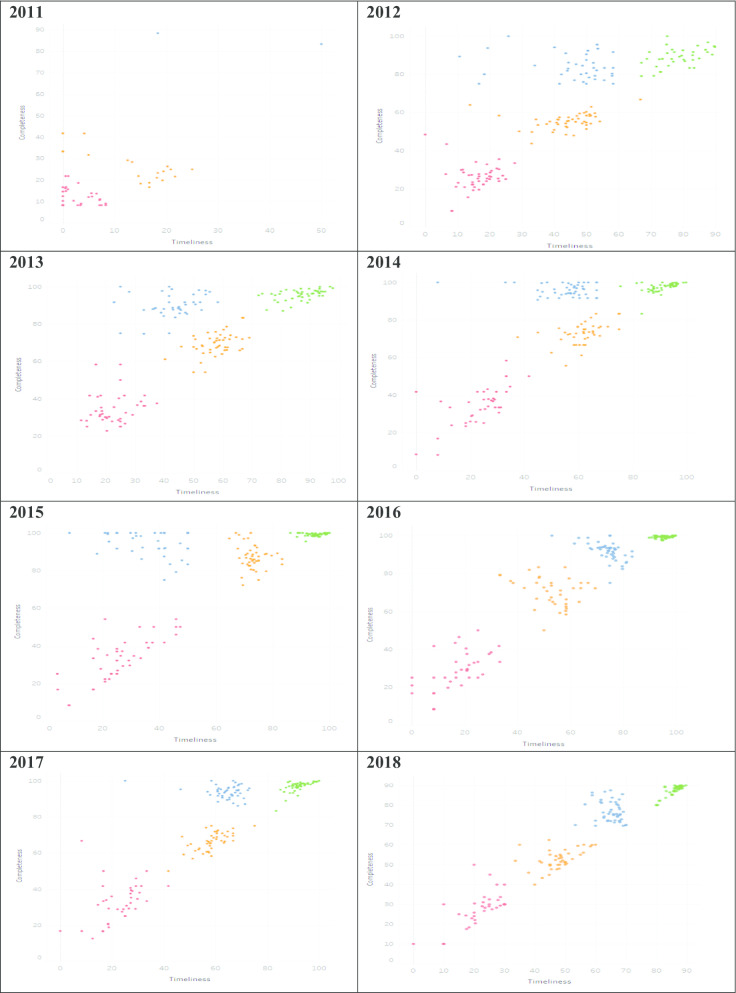
Cluster visualization of facility performance by county illustration for HIV Testing and Counselling

In this section, we present an interactive visual representation of performance cluster groups using scatter charts. As an illustrative example using performance reporting of the HTC programmatic area, Fig. 8 demonstrates the visualization of the average performance of facilities by county for the period 2011 to 2018. Each of the four performance cluster groups are represented using a similar color approach in Figs. 2, 3, 4, 5, 6 and 7. Each point contains the following attributes: name of county, number of facilities represented in that county, and the average completeness and timeliness for the facilities, which are displayed upon hovering the mouse on a point. For example, a green point may represent the average completeness and timeliness for the number of facilities in Nairobi county, which were in the best performing cluster in a particular year. This scenario is replicated for other counties and performance clusters. It is worth noting that facilities represented in each point are of varying characteristics such as type (hospital, health center), and ownership (private, public), hence are clustered based on performance. As such, the points in the scatter chart visualization provide a clear illustration of the four performance cluster groups and their behavior over time. For instance, the initial year of reporting shows only few clusters. Nonetheless, as reporting increases with time, more clusters develop.

Moreover, the outlier performance cluster has shown some improvement in performance as demonstrated with the left movement in the chart over time. The best performing cluster (green) also demonstrates a similar observation with the most improvement in 2016. The illustration in Fig. 2 further shows the proportion of best performing facilities being higher in 2016. Further still, the average facility reporting completeness and timeliness among the average performance cluster group (orange), seemed to have improved in 2015 compared with previous and subsequent years, based on the upward shift in the chart.

## Discussion

The results of our study demonstrate how k-means clustering and interactive cluster-based visualization can be used in identifying patterns and categories within national-level HIV reporting systems, uncovering previously unrecognized patterns. The four categories identified (best performers, average performers, poor performers, and outlier performers) reveal the variation in reporting performance among facilities with respect to year and programmatic area. Moreover, apart from the BS programmatic area, a distinct pattern observed in five of the other programmatic areas was that as the proportion of best performing facilities increased, the proportion of poor performing facilities decreased. In addition, the proportion of facilities in the best performing cluster was higher over time, compared to the proportion of facilities in the other performance clusters. These observations denote improvements in reporting over time within Kenya.

Factors that could explain these improvements in part include data quality improvement procedures done through progressive trainings of those collecting primary data and of health records information officers, provision of technical reporting support to facilities [16]. Other factors such as automation of indicator reporting by electronic medical records (EMRs) to the DHIS2, have the potential to improve routine reporting based on evidence from feasibility studies conducted [29]. With future prospects on automating indicator data reporting, cohort studies can be conducted to establish their impact based on facility reporting completeness and timeliness performance in DHIS2. Further, concerted efforts in improving routine performance of HMIS, touching on technical, behavioral and organizational domains can improve reporting in Kenya [30].

However, despite the observed improvements in performance, there was a decline in proportion of best performing facilities in different years (between 2016 and 2018), depending on the programmatic area. It is worth noting that Kenya experienced one of the longest health worker strike in the public-sector from 5 December 2016 to November 2017, lasting a total of 250 days [31]. The first phase (5 December to 14 March 2017), involved a doctors strike lasting 100 days [31]. Whereas the second phase (5 June to 1 November 2017) involved a nurses strike lasting 150 days [31]. As such, although there may have been other factors that contributed to the decline in proportion of best performing facilities, we suspect that these strikes might have also affected the reporting process. In addition, the decline in 2018 may be attributed to the introduction of new MOH731 summary reporting tools revised in 2018. As such, some facilities were still using the old tool while others had already began using the new tool, signifying the need to improve approaches during transition of reported data.

In overall, we observed that average percentage timeliness tended to be lower compared to average percentage completeness in all the four performance groups. This observation is reflected in other similar studies [12, 32]. Nonetheless, as much as this observation was common among the four performance groups, the outlier performance group specifically brings to light larger disparities between average completeness and timeliness. For instance, as presented in Table 3 for the year 2011, we see that average completeness is 91.67% and timeliness 21.30%. Similar observations can be made for subsequent tables in the various programmatic areas.

Given that timeliness plays an important role in decision-making, there is a cause for concern when there is good effort in submitting of reports, with limitations on timeliness especially in the outlier performance group. As such, there is need for qualitative enquiries to investigate the large disparities in average percentage completeness and timeliness. This is because various factors could act as barriers or facilitators to health facilities ability to attaining and maintaining good completeness and timeliness reporting performance. These factors could be targeted by ministries of health in developing strategies to improve reporting performance of health facilities.

A limitation observed in the scatter chart was that the data points become densely packed in cases where they are many in a small area, hence making it difficult to identify the various points within a cluster. An example is best performers (Fig. 8), more so in 2016. Nonetheless, interactive components (mouse hovering and filtering) incorporated within the scatter chart facilitate access to detailed information. As such, this allows for closer examination of various elements within the data set such as performance in individual counties and number of facilities within a county for a particular performance cluster. This also enables identifying areas that warrant further investigation in their performance, which contributes to informed decision-making. The interactive approach was also used based on the need to visualize various facets of data simultaneously, which can be a challenge [33].

Incorporation of these analyses as well as visualizations to run in real time within aggregate-level HMIS, have the potential to allow monitoring and timely responsiveness to performance changes. Moreover, off shelf software such as Tableau [27], which provide basic modules for free usage can be leveraged as a cost effective alternative for representing and sharing analysis for routinely collected data that has been extracted from large data systems.

The scope of the study can be relevant for many countries dealing with HIV reporting in aggregate-level HMIS. However, the limitation in this study is that data have been collected and analyzed for one country only. Nonetheless, the indicators used (completeness and timeliness) could also be relevant in other contexts. Further, the findings only reflect trends and associations, and do not explain causality. Investigations, including use of qualitative approaches, are needed to definitively determine causes of the observed trends and variations. While we only looked at clustering based on performance, we recognize that performance can be associated with several other factors including facility ownership (private vs public), facility type and level, (for example hospital, dispensary), presence or absence of electronic reporting systems, geographical location and infrastructure availability, among others.

One of the future aims will be to determine factors influencing movement of facilities between clusters with special attention to factors associated with decrease in performance.

## Conclusions

K-means clustering and interactive cluster-based visualization was applied to identify patterns of performance in terms of completeness and timeliness of facility reporting in six HIV programmatic areas. This resulted to four clusters: best performers, average performers, poor performers, and outlier performers, depending on average percentage of completeness and timeliness. The identified clusters revealed general improvements in reporting performance in the various reporting areas over time, but with most noticeable decrease in some programmatic areas between 2016 and 2018. This signifies the need for continuous performance monitoring with possible integration of machine learning and visualization approaches into national HIV reporting systems.

As future work, we will also work with the relevant decision-makers in the study country to incorporate the demonstrated machine learning and visualization approaches for use in automatic and continuous assessment of reporting performance within Kenya.

## Data Availability

The data sets generated during the current study are available in the national District Health Information Software 2 online database, https://hiskenya.org/.

## Abbreviations

ART: Antiretroviral therapy
BS: Blood Safety
CRT: Care and Treatment
DHIS2: District Health Information System Version 2
EMRs: Electronic Medical Record System
HTC: HIV Testing and Counselling
HIV: Human Immunodeficiency Virus
HMIS: Health Information Management Systems
LMICs: Low-middle-income countries
M&E: Monitoring and Evaluation
MoH: Ministry of Health
PEP: Post-Exposure Prophylaxis
PMTCT: Prevention of Mother to Child Transmission
VMMC: Voluntary Medical Male Circumcision
